# Sex Differences in the Effects of Early Life Stressors in a Rat Model of Myofascial Low Back Pain

**DOI:** 10.1002/ejp.70114

**Published:** 2025-08-28

**Authors:** Deepika Singhal, Lin Li, Wolfgang Greffrath, Rolf‐Detlef Treede

**Affiliations:** ^1^ Department of Neurophysiology, Mannheim Centre for Translational Neuroscience, Medical Faculty Mannheim Heidelberg University Mannheim Germany; ^2^ Neuropharmacology Laboratory of Physiology Department Medical School of Nanchang University Nanchang China; ^3^ Department of Psychiatry and Psychotherapy, Central Institute for Mental Health, Medical Faculty Mannheim Heidelberg University Mannheim Germany

**Keywords:** chronic primary musculoskeletal pain, mechanical hyperalgesia, mechanical hypersensitivity, myofascial pain, nerve‐growth factor, paw withdrawal threshold, pressure pain threshold, strain differences

## Abstract

**Background:**

Chronic primary low back pain (cpLBP) is prevalent worldwide. Adverse childhood experiences (ACEs) increase the risk of cpLBP. Here, we explored ACEs as a predisposing factor for adult cpLBP using a rodent model.

**Methods:**

During adolescence, male and female Wistar rats underwent repeated restraint stress (RS) for one hour daily for 12 days or social isolation (SI) for 29 days; controls were handled daily. In adulthood, acute cpLBP was mimicked by NGF or saline injections into the lumbar multifidus muscle. Deep muscular hypersensitivity was assessed using the pressure pain threshold (PPT) of multifidus (MF) and gastrocnemius muscles (GS). Cutaneous mechanical hypersensitivity was measured with paw withdrawal threshold (PWT).

**Results:**

SI had smaller effects than RS in adolescence (*d* = −0.87 vs. −1.20) and adulthood (*d* = −0.33 vs. −0.48). RS and SI had a moderate impact in adolescent females (Cohen's *d* = −0.69), while males experienced a strong sensitisation (*d* = −1.42), with persistence into adulthood only in males (*d* = −0.52). Sensitivity to change was lower for PPT of GS (*d* = −0.71) than PPT of MF (*d* = −1.09) or PWT (*d* = −1.17). PPT of the injected MF dropped in stressed females both to saline (*d* = −0.447) and NGF (*d* = −0.568) but not in males. Both early life stress models induced immediate muscular and cutaneous hypersensitivity that recovered partly in adulthood.

**Conclusions:**

Males were more susceptible to manifest sensitisation by stress than females, whereas females exhibited a memory trace (latent sensitisation), causing hyperalgesia upon the second hit in adulthood. The differential stress sensitivity may contribute to the higher prevalence of cpLBP in females.

**Significance Statement:**

Chronic primary low back pain (cpLBP) is prevalent worldwide, particularly in women. Adverse childhood experiences (ACEs) increase the risk of cpLBP. Using a rat model of cpLBP and two early life stressors, we report that male rats were more susceptible to manifest sensitisation by stress, whereas female rats exhibited a memory trace, causing behavioural signs of hyperalgesia upon the second hit in adulthood (noxious muscle stimulation). The differential stress sensitivity may contribute to the higher prevalence of cpLBP in women.

## 1 Introduction

Chronic primary low back pain (cpLBP), defined by the World Health Organization (WHO) as persisting for at least 3 months without identifiable causes, affects 577.5 million individuals worldwide, resulting in a burden of 57.6 million years lived with disability (Ferreira et al. 2023). This term replaces “non‐specific low back pain”, characterised by chronic discomfort without apparent pathoanatomical causes (Maher et al. 2017). It indicates when chronic pain can be considered a disease rather than a symptom of some other disease (Nicholas et al. 2019). Adverse Childhood Experiences (ACEs) include physical, sexual or emotional abuse and neglect during childhood (Herzog and Schmahl 2018; Jones et al. 2020; Monnat and Chandler 2015). Repercussions of these experiences on the body's stress response system can result in lasting alterations in pain processing (Burke et al. 2017; You et al. 2019). Moreover, there are sex‐specific differences in how stress influences the prevalence and response to pain (Green et al. 2021; Grégoire et al. 2020; Louwies and Greenwood‐Van Meerveld 2020; Weiß et al. 2023).

ACEs can be modelled by early‐life stressors in animals. In animal models, stress has been found to have a pivotal role in modulating pain perception and intensity (Gregory et al. 2013; Patchev et al. 2006; Tay and Grundy 2023). In humans, ACEs have been linked to cpLBP in adulthood, with emotion regulation difficulties mediating this relationship (Thomas and Goodin 2022).

Nerve growth factor (NGF) is a key player in signalling soft tissue (muscle) injury (Deising et al. 2012). Repeated injection of low‐dose NGF has been established as a rat model of myofascial cpLBP (Hoheisel et al. 2013, 2015; Reed et al. 2020, 2022; Sessler et al. 2021; Singaravelu et al. 2021; Syrett et al. 2022; Zhang et al. 2017): a first conditioning injection leaves a memory trace (latent sensitisation), so that a second injection has prolonged efficacy (manifest sensitisation). Stress experienced during adulthood can sensitise dorsal horn neurons (DHNs) in the rat spinal cord, intensifying responses to painful stimuli upon subsequent NGF exposure (Hoheisel et al. 2015; Singaravelu et al. 2021). Stress experienced during adolescence can also cause mechanical hypersensitivity, which is persistent till adulthood (Singaravelu et al. 2022).

We aim to explore sex differences in the effects of adolescent stress on behavioural signs of heightened sensitivity of dorsal horn neurons (DHNs), potentially leading to cpLBP in adulthood. Previous animal studies were mostly done in males (Hoheisel et al. 2007; Singaravelu et al. 2021; Zhang et al. 2017), while clinically, females are affected more frequently (Bartley and Fillingim 2013). Furthermore, we seek to investigate how two prototypical types of stressors influence the intensity of cpLBP. To achieve this, we compared repeated restraint stress (RS) to mimic physical trauma and social isolation (SI) to replicate emotional neglect in animal stress models. In adherence to the principles of 3Rs (Replace, Reduce, Refine), we re‐used PPT and PWT data from a recently published study conducted in our lab under identical conditions on male Wistar rats in one of the stress models (RS) (Singaravelu et al. 2022).

## 2 Methods

The study adheres to the ARRIVE guidelines (see Table 1). All experiments were performed in compliance with the German law on the protection of animals under the permission number: QZ 35‐9185.81/G‐7/19. Sixty female and thirty male Wistar HAN rats were used in the study; data from an additional 24 male rats from a previous study (Singaravelu et al. 2022) were re‐analysed. We investigated restraint stress and social isolation stress paradigms in both sexes for a sex difference analysis.

**TABLE 1:** ARRIVE guideline: A checklist of 10 essential checklist items for animal studies.

**FIGURE 1:**
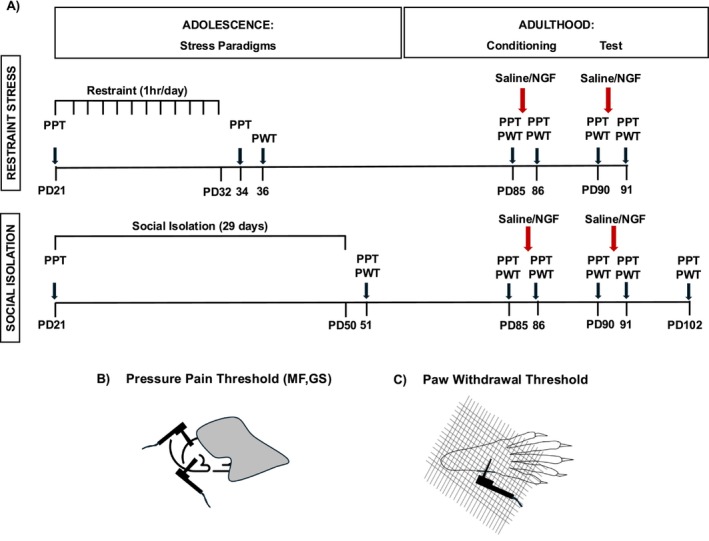
Experimental design for comparing effects of early life stressors (restraint or social isolation) on the myofascial low back pain model. (A) The experimental procedure was divided into two phases: adolescence and adulthood. In adolescence, animals were stressed under two paradigms: restraint stress (RS) or social isolation (SI); controls were handled. RS groups received stress for 12 consecutive days 1 h each day (from PD21‐32). SI groups were kept single‐housed for 29 days (PD21‐50). Behavioural tests for evoked pain sensitivity were done before (PD21) and immediately after stress (PD34/PD36 or PD51). In adulthood, animals received two injections on PD85 (conditioning) and PD90 (test) with either NGF or Saline (represented by downward black arrows). The behavioural tests for evoked pain sensitivity were recorded before (PD85, PD90) and after the injections (PD86, PD91); see the Methods section for combinations of stressor/1st/2nd injection that were tested according to the hypotheses. (B) Illustration of the PPT procedure: the animal was covered with a towel, and the algometer was used to measure the PPT of the MF and GS muscles with a blunt 2 mm diameter tip. (C) Illustration of the PWT procedure: the left hind paw of the animal on the grid, and the PWT was measured by the algometer with a 0.8 mm diameter tip. NGF, nerve growth factor (0.8 μM, 50 μL); PD, postnatal day (age of animal); PPT‐GS, pressure pain threshold for deep pain sensitivity of the gastrocnemius muscle; PPT‐MF, pressure pain threshold for deep pain sensitivity of the low back multifidus muscle; PWT, paw withdrawal threshold for cutaneous pain sensitivity of the distal hind paw.

1. Study design
Longitudinal study and parallel group design (Methods, Section 2) The experimental unit is each animal
2. Sample size
A total of 60 female and 30 male Wistar rats were used (Details in Section 2.1). The data from an additional 24 male Wistar rats (from Singaravelu et al. 2022) was used for the sex difference comparison. Sample size and experimental procedure approved by “Regierungspräsidium”, the regional board, Karlsruhe, Germany A total of 114 animals were used. Data from 112 were analysed (one male and one female died on PD23 and PD24, respectively, during the restraint stress paradigm) Sample size was based on Singaravelu et al. (2022). Stressed and controls were always studied together. 6 cohorts in females and 6 cohorts in males in a group of 4 animals for restraint stress and a negative control cohort of 6 animals (CSS). For SI, 2 cohorts in males and 2 cohorts in females in groups of 15 animals each
3. Inclusion and exclusion criteria
No strict inclusion and exclusion criteria One stressed female (from Stress + Saline + NGF group in restraint stress) was excluded, as it died on PD24. Likewise, one stressed male was excluded as it died on PD23 N has been mentioned in Section 2.1 in methods
4. Randomisation
No randomisation was used Identical protocol followed for all treatments to minimise the investigator's bias
5. Blinding/Masking
The experimenter (D.S.) was blinded for the assignment of the animals to the treatment groups and the recordings of the behavioural measurements. L.L. had access to the code for unblinding and took notes during the experiment, but did not communicate with D.S
6. Outcome measures
Blunt pressure pain threshold of the multifidus muscle (PPT‐MF) (local) and gastrocnemius muscle (PPT‐GS) (remote) using 2 mm diameter (cut‐off: 600 g), punctate stimuli paw withdrawal threshold (PWT) (remote) using 0.8 mm diameter (cut‐off: 100 g) PPT of the multifidus muscle
7. Statistical methods
Mann–Whitney *U*‐test. Cohen's *d*. Forrest plots for within‐study meta‐analysis (Methods Section 2.7)
8. Experimental animals
Adolescent male and female rats (Wistar Han outbred), initial body weight 50 g. Terminal body weight 200–225 g (females) and 385–415 g (males) The animals were also monitored for the grooming behaviour and abnormal posture
9. Experimental procedures
Experimental procedure: stress paradigms described in Section 2.2, nerve growth factor or saline injections in Section 2.3. Treatment groups in Section 2.4 and behavioural tests in Section 2.5. The timeline of the procedure is highlighted in Figure 1. All experimental procedures were done in the same room. The rationale has been mentioned in the Introduction (Section 1)
10. Results
Data are given as means ± SE; forest plots (in Figures 4, 5, 6) represent comparisons across sexes, stress states and different readouts (PPT, PWT). The details mentioned in Results (Section 3)

### 2.1 Animals

Animals were sourced from ENVIGO in the Netherlands at postnatal day 21. Upon arrival, they were housed in groups of four for the restraint stress study and five for the social isolation study, within standard Makrolon cages measuring 55 × 35 × 20 centimetres. The animals were provided ad libitum access to food and water and were maintained under a standard 12‐h light/dark cycle. Following a brief acclimatisation period of approximately 2 h in the new cage and with their cagemates, the animals were transferred to experimental procedure rooms in their home cage. They were allowed an additional 1‐h acclimatisation period. The same experimental room was used for stress and behaviour assessment. Before the behaviour measurement (PPT), the heads of the animals were covered with a cloth for 10 min. All experiments were conducted during the animals' inactive phase. The same experimenter (DS) consistently handled animals throughout the study. The experimenter was also blinded throughout the study.

### 2.2 Stress Paradigms

In this study, the animals were stressed during adolescence, which lasts from PD21 to around PD60 (Arellano et al. 2024; Cottam et al. 2025). During the adolescent phase, 10.5 rat days are equivalent to one human year (Sengupta 2013).

Restraint Stress: The stress paradigm was conducted in the early adolescent phase. The female Wistar rats were kept inside a narrow cylindrical restrainer (inner length 15 cm; inner height 4 cm) for 1 h daily for 12 consecutive days. The animals were stressed from PD21 to PD34. The control animals were handled by the same experimenter (DS). Each cage consisted of four animals, of which two were stressed and two were controls. The present study transfers a previous study in male animals (Singaravelu et al. 2022) to females; for sex differences, these two data sets are compared. Body weight was measured every day before beginning the stress paradigm. In each cage, there were four animals, two stressed and two controls.

Social Isolation: An acute early life social isolation, followed by regrouping, was done in the adolescent phase, starting from PD21. While long‐term social isolation is considered a severe stressor for social animals (Lavenda‐Grosberg et al. 2022), we employed transient isolation, which is much less severe. Previous studies had shown that this paradigm of acute social isolation is sufficient to induce significant alterations in several behavioural tests in adulthood (Graf et al. 2024). Both female and male Wistar rats were kept single‐housed in standard Makrolon cages for 29 consecutive days (Maslova et al. 2010). Before separation, five animals were habituated together in the cage, and post‐habituation, two animals were randomly picked and socially isolated in separate cages. The control animals were kept in a group of three in their home cages. On PD51, the animals were regrouped with their respective cages, resulting in five animals per cage (two stressed and three controls).

### 2.3 Myofascial Low Back Pain Model

In adulthood, some non‐stressed animals were injected with saline or NGF in the left multifidus muscle at an interval of 5 days (on PD85 and PD90) to reproduce the myofascial low back pain model previously established in Sprague–Dawley rats (Hoheisel et al. 2013). In this model, the first NGF injection (conditioning) induces a memory trace in dorsal horn neurons (latent sensitisation) such that the threshold decreases in response to the second NGF injection (test) are enhanced and prolonged. Stressed animals received NGF only as the second injection in order to assess if the stress paradigms in adolescence had left a memory trace (latent sensitisation) into adulthood.

Nerve Growth Factor (NGF, 0.8 μM human recombinant, Calbiochem, MERCK, Germany) was dissolved in phosphate buffer saline (PBS: pH of the NGF solution 7.2–7.3). The amount of 50 μL (NGF/Saline) was administered into the left multifidus muscle, located 3 mm lateral to the spinous process at vertebral level L5. This concentration of NGF did not cause immediate ongoing pain but induced hyperalgesia, which can be classified as evoked pain (Deising et al. 2012) when injected into the muscles of humans. Saline injections served as the control, with no observed signs of muscle inflammation, as reported in previous studies (Hoheisel et al. 2013; Hoheisel and Mense 2015; Sessler et al. 2021; Singaravelu et al. 2021, 2022; Zhang et al. 2017). This model demonstrates face validity as a model of hyperalgesia. In rats, PPT and PWT are used to assess mechanical hyperalgesia, compared to quantitative sensory testing (QST) in humans (Stojanovic 1998). Treatment efficacy of physiotherapy in humans (Dove et al. 2023) and spinal manipulation (Reed et al. 2022) and exercise in rats (de Azambuja et al. 2018) underline the predictive validity of the model.

### 2.4 Treatment Groups

To explore sex differences in latent sensitisation by two adolescent stressors, we applied interventions at three time points (Figure 1A): stress during adolescence, followed by two injections in adulthood; first as conditioning (PD85) and second as test stimulus (PD90). The stress paradigms were designed to mimic childhood adversities in humans and were hypothesised to leave a memory trace (latent sensitisation). The first (conditioning) NGF injection was designed to mimic a preceding mild muscle injury and was also hypothesised to induce a latent sensitisation. The second NGF injection was used as mild nociceptive input to test if either stress or conditioning NGF had induced a latent sensitisation.

The treatment groups were treated as follows: each treatment group consisted of six animals.

- Stress + Saline +NGF (SSN): This is the main experimental group that was used to study if stress could cause a latent sensitisation of the DHNs. Hence, the animals were stressed in adolescence, and Saline was injected on PD85 followed by NGF on PD90 (as the nociceptive input in adulthood).
- Control + Saline + NGF (CSN): This is the control group for effects of NGF without preceding latent sensitisation. Non‐stressed animals are administered with NGF only on PD90.
- Stress + Saline + Saline (SSS): This is the control group for the effects of stress without NGF. Animals experienced stress during adolescence, and two saline injections were administered on PD85 and PD90.
- Control + NGF + NGF (CNN): This is the positive control group where non‐stressed animals are injected with two NGF injections consecutively, one for the induction of latent sensitisation; the second for testing the presence of latent sensitisation (Hoheisel et al. 2013).
- Control + Saline + Saline (CSS): This is the negative control group for the positive control (CNN), where non‐stressed animals are injected with two saline injections consecutively on PD85 and PD90.

A full factorial design with three intervention time points would have included a total of 2 × 2 × 2 = 8 groups. The following three groups were not studied according to the 3R principle because they did not serve to test any existing hypothesis:

- SNS (Stress + NGF + Saline): This would have been an alternative main experimental group to study if stress could cause a latent sensitisation of the DHNs. Here, NGF would have been injected on PD85 instead of on PD90. CNS (Control + NGF + Saline) would be the respective control for the effects of NGF without preceding latent sensitisation. This design would not have included a positive control for the responsiveness of the Wistar rats to latent sensitisation in a validated paradigm established in SD rats (Hoheisel et al. 2013; Reed et al. 2020)
- SNN (Stress + NGF + NGF): Would test an additive effect of latent sensitisation by adolescent stress plus mild muscle injury in adulthood (first NGF) on responses to second NGF.

For social isolation stress (SI), animals were ordered in cohorts of 15 (2 male and 2 female cohorts), divided into 3 cages with 5 animals each. In each cage, 2 animals were socially isolated and later returned to their original cage. Each cage included one animal from each experimental group: (SSS, CSS), (SSN, CSN) and CNN. Behavioural testing was conducted on the same day for the entire cohort. As a limitation, we cannot exclude the social transfer of learned stress behaviour (Smith et al. 2021).

For restraint stress (RS), animals were ordered weekly in cohorts of 4 (6) female cohorts in this study; 6 male cohorts in (Singaravelu et al. 2022). All 4 animals were housed together; 2 were stressed and returned, while 2 were only handled. Each cage included one animal from each group: (SSS, SSN), (CSN, CNN). Behavioural testing occurred on the same day per cage. The CSS control group (no stress, no NGF) was studied separately in 6 female animals across 2 cages, as this group was identified as missing in the previous male study (Singaravelu et al. 2022).

### 2.5 Behavioural Test

Behavioural tests were conducted to evaluate signs of mechanical hyperalgesia in low back and calf muscles (pressure pain threshold) and in hind paw skin (paw withdrawal threshold). Before the behavioural tests, the animals were habituated to the experiment room for 1 h for the baseline test.

Pressure pain threshold of the low back multifidus muscle:

To assess local mechanical hypersensitivity, the pressure pain threshold (PPT) of the low back multifidus muscle was measured using an electronic Von Frey aesthesiometer (IITC 2390 series, Life Science Instruments, Woodland Hills, CA, USA) with a blunt polypropylene probe (2 mm diameter, cut‐off force 600 g). The animal was covered with a towel, and the algometer was used to apply pressure with a steadily increasing force (Reitz et al. 2016) perpendicular to the multifidus muscle through intact skin at vertebral level L5 (Figure 1B). It activates mainly the nociceptors from deep tissue, and the nociceptors from the skin are only marginally excited (Nasu et al. 2010; Takahashi et al. 2005). The PPT was the minimum pressure intensity required to elicit a pain‐related reaction (vocalisation, withdrawal or escape movements). PPT measurements were taken before and after stress and in conjunction with intramuscular injections. The PPT measurements were conducted once per animal per time point to avoid the habituation of the animals to the minimum pressure intensity. This paradigm is similar to PPT measurement on the low back in humans (Pfau et al. 2014), but behavioural thresholds in rats are somewhat lower than pain thresholds in humans (Reitz et al. 2016).

Pressure pain threshold of the gastrocnemius muscle:

Like the PPT‐MF, the pressure pain threshold of the GS muscle was measured to evaluate the remote mechanical hypersensitivity using the electronic Von Frey aesthesiometer with the blunt polypropylene probe (2 mm diameter). The pressure was applied with increasing intensity to the GS muscle on the thigh through intact skin (Figure 1B). The PPT was the minimum pressure intensity required to elicit a pain‐related reaction (vocalisation, withdrawal or escape movements). PPT measurements were taken before and after stress and in conjunction with intramuscular injections. The PPT measurements of the GS muscle were conducted once per animal per time point to avoid the habituation of the animals to the minimum pressure intensity.

Paw withdrawal threshold of the left hind paw:

To evaluate cutaneous mechanical hypersensitivity, the paw withdrawal threshold (PWT) to punctate mechanical stimuli was assessed in both distal hind limbs following PPT measurements using a cylindrical probe (0.8 mm diameter, cut‐off force 100 g). The animals were habituated in a Plexiglas box with a metal grid base before applying increasing punctate pressure to the plantar region of the left hind paw (Figure 1C). Before the first measurement, the animals were habituated to the metal grid table for two consecutive days before the test, including on the test day. The PWT measurements were the arithmetic mean of 5 suprathresholds for the RS stress paradigm (for both males and females) and 3 suprathresholds for the SI stress paradigm (for both males and females). This paradigm is similar to pinprick pain threshold measurement on the dorsum of the foot in humans (Pfau et al. 2014), but behavioural thresholds in rats are somewhat lower than pain thresholds in humans (Reitz et al. 2016).

The behavioural tests had previously demonstrated the preventive effects of exercise, suggesting that they are sensitive to change (de Azambuja et al. 2018; Hoheisel et al. 2013).

### 2.6 Timeline

Upon arrival, baseline PPT was obtained on PD21. Animals underwent one of the two stress paradigms or the respective control conditions. Subsequently, PPT was obtained again post‐stress on PD34 (for RS) and on PD51 (for SI). Due to timing constraints, PWT was only determined after the stress or control conditions. All animals then received two injections of either NGF or saline on PD85 (conditioning stimulus) and PD90 (test stimulus). PPT and PWT were measured immediately before the injection and 1 day later (PD86, PD91). In SI, the PPT and PWT were additionally measured on PD102 (12 days post‐second injection) (Figure 1A).

The animals were weighed upon arrival and before the pain measurements on PD34 (RS), PD51 (SI) and PD85 (adulthood). There was no significant growth retardation into adulthood following the adolescent stress paradigms; only a transient retardation in female rats subjected to restraint stress (Table 2).

**TABLE 2:** The evolution of body weight in animals from before the stress paradigm to adulthood.

Time points	Stress	Sex	Mean ± SD (Stress)	Mean ± SD (Controls)	*p*	Cohen's *d*
Before stress or handling (PD21)	RS	Female	49.5 ± 03.7	49.1 ± 3.2	0.799	0.100
	RS	Male	50.7 ± 05.3	51.1 ± 5.8	0.965	0.026
	SI	Female	45.1 ± 02.7	44.3 ± 4.2	0.415	0.297
	SI	Male	50.0 ± 00.0	48.8 ± 2.1	0.129	0.278
End of stress or handling (PD34/PD51)	RS	Female	103.6 ± 05.9	112.5 ± 08.6	0.024*	1.236###
	RS	Male	127.6 ± 09.6	129.7 ± 13.3	0.945	0.129
	SI	Female	157.5 ± 10.9	153.8 ± 09.6	0.998	0.337#
	SI	Male	230.0 ± 16.2	235.2 ± 14.9	0.896	0.468#
Adulthood (PD85)	RS	Female	205.8 ± 06.8	215.5 ± 20.2	0.804	0.620##
	RS	Male	393.1 ± 37.7	396.6 ± 34.4	0.985	0.013
	SI	Female	219.9 ± 18.0	214.7 ± 12.0	0.999	0.171
	SI	Male	391.2 ± 28.3	388.0 ± 25.5	0.995	0.562##

*Note:* The table represents the body weight of animals (stress and controls) measured in grams, before stress paradigms or handling of controls (PD21); at the end of the stress paradigm or handling in adolescence (PD34/PD51) and in adulthood (PD85). The body weight is presented as Mean ± SD. Cohen's *d* has been calculated using the Mann–Whitney *U*‐test statistics (Lenhard and Lenhard 2022). The *p*‐value is calculated using the Mann–Whitney *U*‐test: *p < 0.05. Effect sizes according to Cohen's *d*; ###: *d* > 0.8 refers to a large effect, ##: 0.8 > *d* > 0.5 refers to a medium effect, #: 0.5 > *d* > 0.2 refers to a small effect.

Abbreviations: RS, Restraint stress; SI, Social isolation.

### 2.7 Data Analysis

A total of 114 animals entered the experimental paradigm. However, one female rat died on day 4 and one male rat died on day 3 of the restraint stress paradigm. Both were from the SSN groups and were excluded from further analyses, leaving a total of 112 animals for analyses (see also Table 1).

Statistical analyses were conducted using Microsoft Excel (version 16.90.2), GraphPad Prism (version 10) and R Studio (version 4.3.2). A normal distribution of the data was tested with the Kolmogorov–Smirnov test. Most data did not fit the rules of parametric analysis; hence, all comparisons were performed with the Mann–Whitney *U* test. Statistical significance was considered with an alpha level of 0.05 or lower (*p* < 0.05, two‐tailed). All values were log‐transformed using base 2 before the analysis.

Because the priming effects of the clinically relevant stressor, social isolation, had not been studied before, our analysis approach was primarily exploratory. We therefore report effect sizes for all salient comparisons. Effect sizes were calculated between groups using Cohen's d as the mean difference divided by the pooled SD. For non‐normally distributed data, the test statistics of the Mann–Whitney *U* test (calculated by GraphPad Prism) were used to calculate *η* ^2^ = (*Z*/√*N*), where *Z* is the *Z*‐score from the Mann–Whitney *U* test and *N* is the total sample size. Then, appropriately transformed to Cohen's d by using d = 2√*η* ^2^/√(1 − *η* ^2^), to quantify the magnitude of differences observed (Fritz et al. 2012; Lenhard and Lenhard 2022). Interpretation of effect sizes was (cf. Cohen papers) as follows: small effect size *D* > 0.2, medium effect size *D* > 0.5, significant effect size *D* > 0.8. Effect sizes will enable power calculations for future studies on the prevention and treatment of low back pain in animal stress models.

Availability of standardised effect sizes (and 95% confidence intervals) for all paired comparisons between stressed and matched control groups permitted the running of within‐study meta‐analyses to assess the influence of sex, stressor and behavioural readout. A random‐effects (RE) model was used to pool effect size estimates across treatment groups and/or readouts, accounting for heterogeneity across contrasts forming a “nested outcome”. Results were visualised using a forest plot, which allows for a clear interpretation of individual and overall effects, including their direction, magnitude and precision (Soliman et al. 2021; Vesterinen et al. 2014).

## 3 Results

Most behavioural readouts changed over time from PD21 to PD85. We interpret these trends as signs of maturation of the nociceptive system; however, residual effects from previous transport stress may have also contributed. To compensate for these time‐dependent trends, all analyses on the effects of stress were done by comparison with the matched control groups that were only handled.

### 3.1 Deep Muscular and Cutaneous Hypersensitivity Induced by Restraint Stress (RS)

**FIGURE 2:**
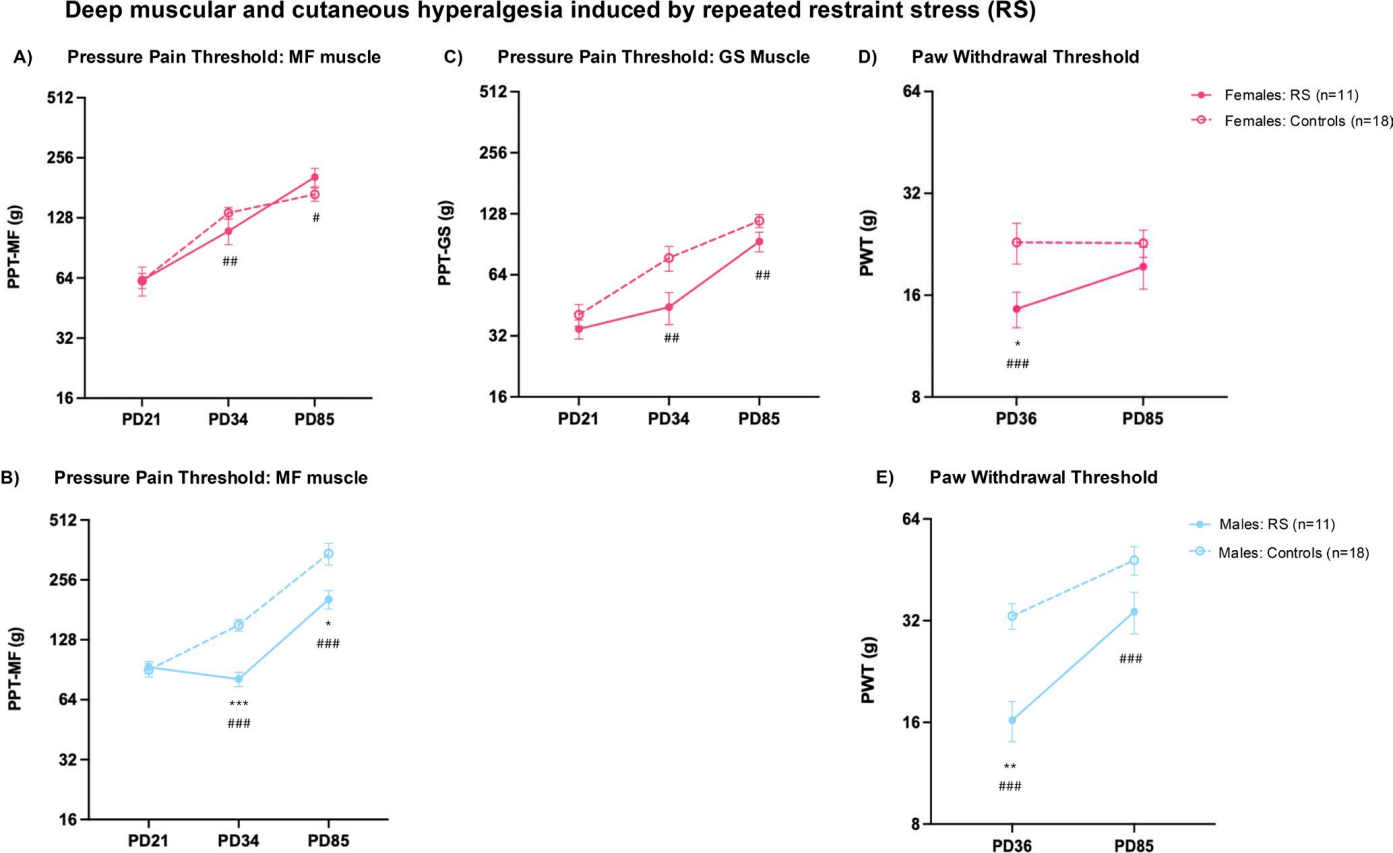
Deep muscular and cutaneous hyperalgesia induced by repeated restraint stress. Left: Mean PPT of the low back multifidus muscle (MF) as a pain‐related behavioural reaction before (PD21) and after restraint stress (RS) in adolescence (PD34) and in adulthood (PD85) in females (A) and in males (B). Since these data were obtained before the first injection, they are pooled across injection paradigms. Centre: Mean PPT of the gastrocnemius muscle (GS) as a deep muscular pain‐related behavioural reaction before RS (PD21), in adolescence (PD34) and in adulthood (PD85) in females (C). Right: Mean PWT of the left hind paw as a remote pain‐related behavioural reaction post‐RS in adolescence (PD36) and in adulthood (PD85) in females (D) and in males (E). Data is reported as mean ± SEM. The data from males (both stressed and controls) has been obtained from Singaravelu et al. (2022). PD, postnatal day; PPT, pressure pain threshold; PWT, paw withdrawal threshold. Significance according to the *U*‐test of Mann and Whitney: *p < 0.05, **p < 0.01, ***p < 0.001. Effect sizes according to Cohen's *d*: ###: *d* > 0.8 refers to a large effect, ##: 0.8 > *d* > 0.5 refers to a medium effect, #: 0.5 > *d* > 0.2 small effect.

On PD21, there was no difference in deep pain sensitivity between the stress and control groups in both male and female rats (PPT of the lower back MF muscle, Figure 2A,B; PPT of the thigh GS muscle, Figure 2C). Upon repeated RS in females, the PPT‐MF dropped below the control group with a medium effect size but without significance (*p* > 0.05, Cohen's *d* = −0.50, Figure 2A). In adulthood (PD85), the females recovered (*p* > 0.05, Cohen's *d* = +0.463 vs. control group). Males showed a large and significant drop in the PPT‐MF below the control group on PD34 (*p* < 0.001, Cohen's *d* = −2.414, Figure 2B), suggesting that the RS stress paradigm induced axial muscle hyperalgesia that was persistent till PD85 in adulthood (*p* < 0.05, Cohen's *d* = −1.137, male data from (Singaravelu et al. 2022)).

PPT of the GS muscle was measured only in female animals. A medium but non‐significant drop in PPT‐GS was found after repeated RS on PD34 (*p* > 0.05, Cohen's *d* = −0.749), which persisted till PD85 (*p* > 0.05, Cohen's *d* = −0.689) (Figure 2C).

Paw withdrawal threshold (PWT, cutaneous pain sensitivity, Figure 2D) on PD36 in females was significantly reduced with a large effect size (*p* < 0.05, Cohen's *d* = −0.896). On PD85, the drop in threshold was small and non‐significant (*p* > 0.05, Cohen's *d* = −0.244). In males, the drop in PWT on PD36 had a large effect size and was significant (*p* < 0.01, Cohen's *d* = −1.792) that was partly persistent into adulthood with a still large effect size, but not significant (*p* > 0.05, Cohen's *d* = −0.901) (Figure 2E; male data from Singaravelu et al. (2022)).

Hence, RS had medium to large effects on deep muscular pain sensitivity (MF or GS muscles) as well as cutaneous pain sensitivity (PWT) in males and females, which persisted into adulthood in males but less so in females.

### 3.2 Deep Muscular and Cutaneous Hypersensitivity Induced by Social Isolation Stress (SI)

**FIGURE 3:**
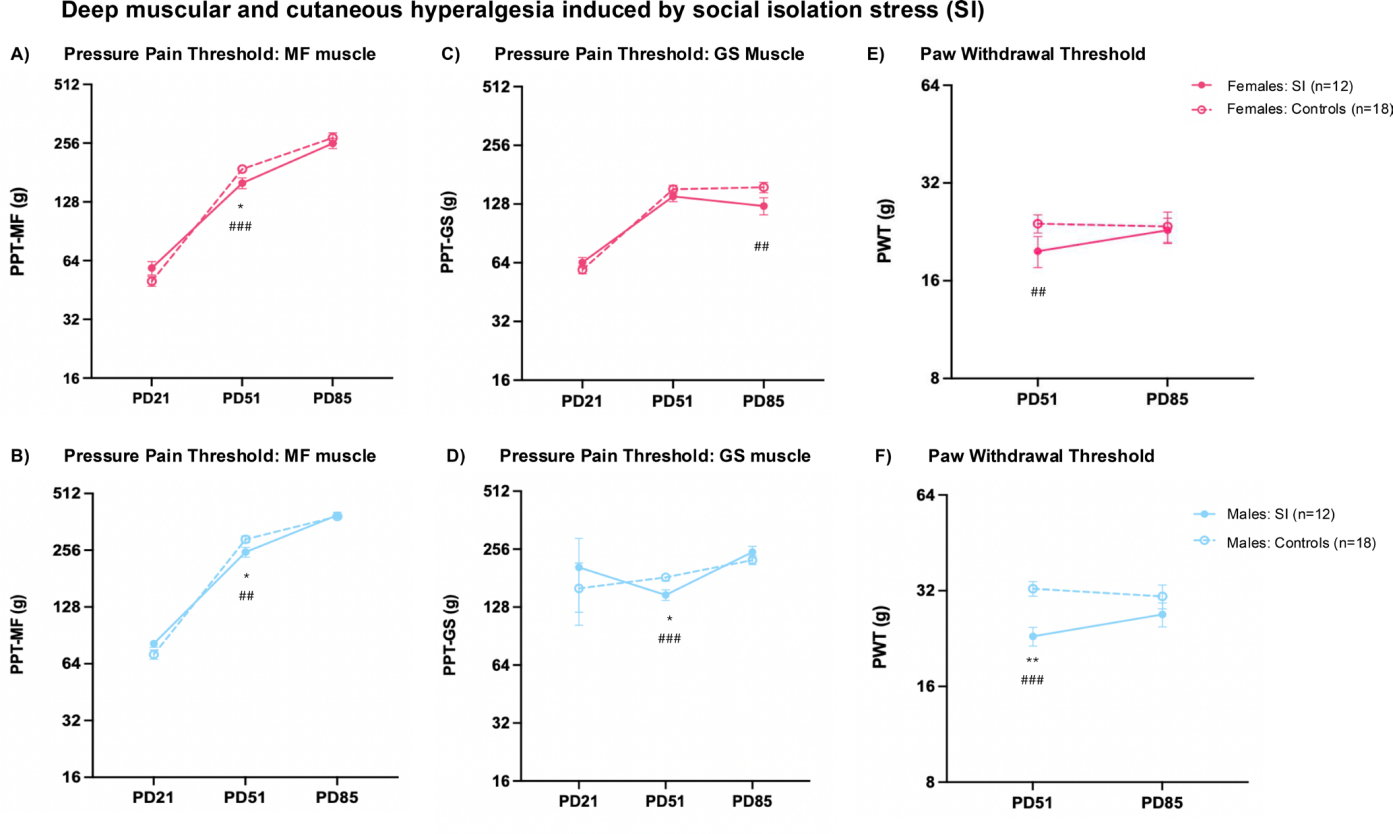
Deep muscular and cutaneous hyperalgesia induced by social isolation stress. Left: Mean PPT of the low back multifidus muscle (MF) as a pain‐related behavioural reaction before (PD21) and after social isolation (SI) in adolescence (PD51) and in adulthood (PD85) in females (A) and in males (B). Since these data were obtained before the first injection, they are pooled across injection paradigms. Centre: Mean PPT of the gastrocnemius muscle as a deep muscular pain‐related behavioural reaction before SI (PD21) in adolescence (PD51) and in adulthood (PD85) in females (C) and males (D). Right: Mean PWT of the left hind paw post‐SI in adolescence (PD51) and in adulthood (PD85) in females (E) and in males (F). Data is reported as mean ± SEM. PD, postnatal day; PPT, pressure pain threshold; PWT, paw withdrawal threshold. Significance according to the *U*‐test of Mann and Whitney: *p < 0.05, **p < 0.01, ***p < 0.001. Effect sizes according to Cohen's *d*: ###: *d* > 0.8 refers to a large effect, ##: 0.8 > *d* > 0.5 refers to a medium effect, #: 0.5 > *d* > 0.2 small effect.

On PD21, there was no difference in deep pain sensitivity (PPT of the lower back MF muscle, Figure 3A,B; PPT of calf GS muscle, Figure 3C,D) between the stress and control groups in both male and female rats. Immediately after SI, females had a significant reduction with large effect size in the PPT‐MF on PD51 (*p* < 0.05, Cohen's *d* = −0.96, Figure 3A). Males also showed a significant drop in the PPT‐MF with medium effect size (*p* < 0.05, Cohen's *d* = −0.78, Figure 3C). However, the hyperalgesia was not persistent into adulthood, neither in females (*p* > 0.05, Cohen's *d* = −0.202) nor in males (*p* > 0.05, Cohen's *d* = −0.171) (Figure 3A,B).

The PPT of the GS muscle was measured in both males and females. In females, a small non‐significant reduction in the PPT‐GS was observed directly post‐SI (*p* > 0.05, Cohen's *d* = −0.378), and this turned into a medium effect‐size non‐significant reduction in adulthood (*p* > 0.05, Cohen's *d* = −0.761, Figure 3C). In males, there was a significant reduction in PPT‐GS with a large effect size immediately after SI (*p* < 0.05, Cohen's *d* = −1.025); but this hyperalgesia did not persist into adulthood (*p* > 0.05, Cohen's *d* = −0.329, Figure 3D).

Cutaneous pain hypersensitivity immediately after SI in females was non‐significant but with a medium effect size (*p* > 0.05, Cohen's *d* = 0.705); whereas in adulthood, the reduction in PWT was negligible (*p* > 0.05, Cohen's *d* = −0.178) (Figure 3E). Cutaneous pain hypersensitivity was significant in males with large effect sizes immediately after SI (*p* < 0.01, Cohen's *d* = −1.511) with partial recovery in adulthood (*p* > 0.05, Cohen's *d* = −0.345, Figure 3F).

Hence, SI had medium to large effects on deep muscular pain (MF or GS muscles) as well as cutaneous pain sensitivity (PWT) in males and females, which recovered in adulthood.

### 3.3 Sex Differences in Mechanically Evoked Nociceptive Signalling

**FIGURE 4:**
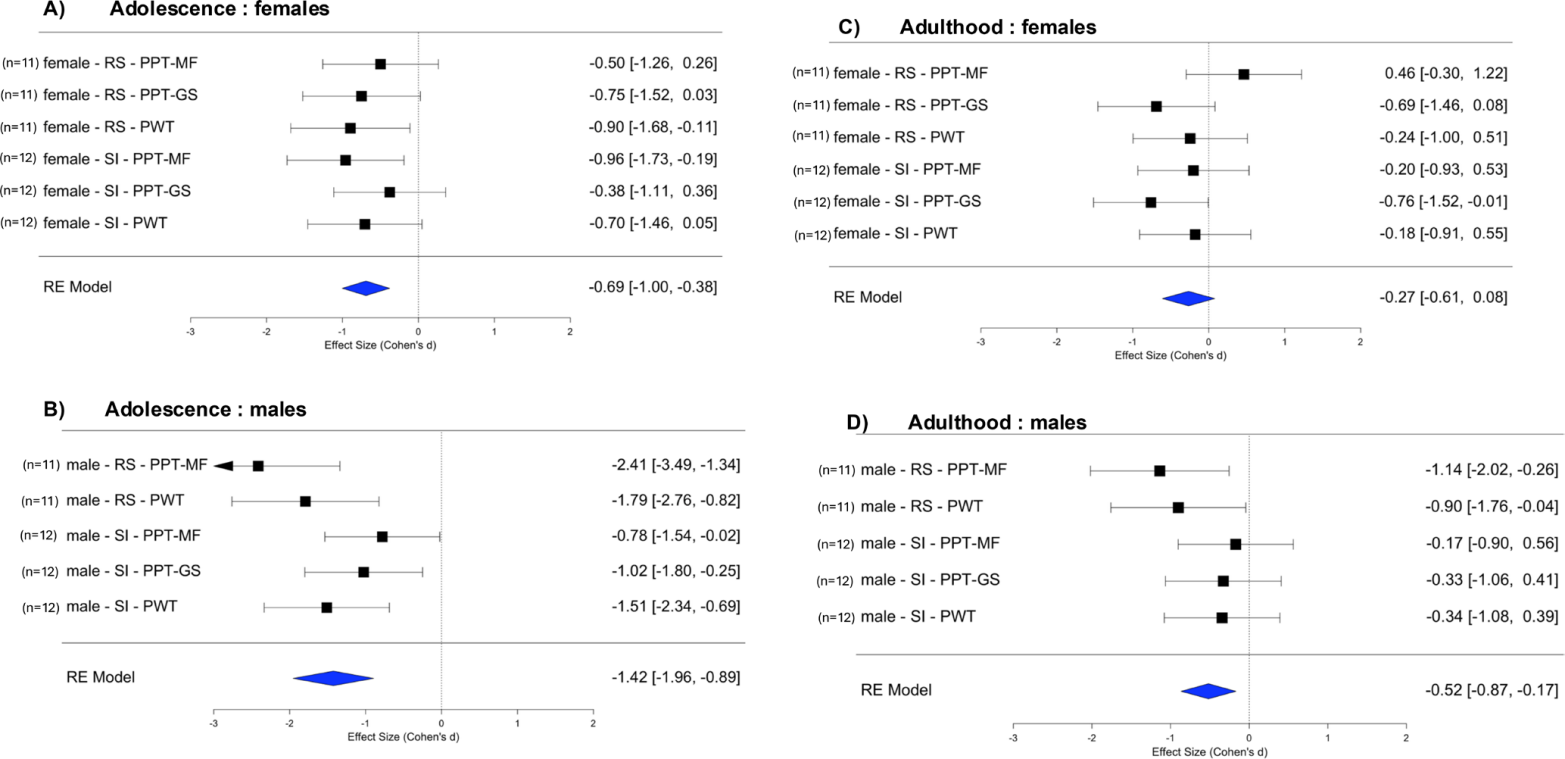
Comparison of effects of early life stress in female vs. male rats. Forest plots of standardised effect sizes with 95% confidence intervals across stressors and readouts (diamonds). Squares and error bars provide effect sizes calculated between stressed and respective control animal groups. Adolescence: immediately after stress (A. females, B. males). Adulthood: after puberty, immediately before injections of NGF or saline (C. females, D. males). The male‐RS data has been obtained from Singaravelu et al. (2022). NGF, nerve growth factor (0.8 μM, 50 μL); PPT‐GS, pressure pain threshold of the lower limb gastrocnemius muscle; PPT‐MF, pressure pain threshold of the low back multifidus muscle; PWT, paw withdrawal threshold to cutaneous pin prick; RS, restraint stress; SI, social isolation stress.

Females were generally more sensitive than males to stimuli that evoke deep muscle pain. In the non‐stressed control groups, this difference persisted throughout the lifespan assessed here (compare dashed lines in Figure 2A vs. B, Figure 3A vs. B). Females were also more sensitive to stimuli that evoke cutaneous pinprick pain (PWT, compare dashed lines in Figure 2D vs. E, Figure 3E vs. F), for which baseline data were not available. Sex differences in the effects of the two stressors (repeated restraint RS, social isolation SI) were therefore evaluated using within‐study meta‐analyses of standardised effect sizes of the stressed group vs. the respective control group (see Forest plots, Figure 4).

Combined across both stressors and all three readouts, the effect size in females was medium in adolescence (mean *d* = −0.69, Figure 4A), whereas in males, the effect size was large (mean *d* = −1.42, Figure 4B). In contrast, in adulthood, the effect size in females was small (mean *d* = −0.27, Figure 4C) and in males was medium (mean *d* = −0.52, Figure 4D). If the 95% CI for a mean effect size contains zero, it suggests statistical non‐significance (Lee 2016). Hence, 95% CIs of the pooled data revealed significant manifest sensitisation in both sexes during adolescence, which decreased but was still significant in males in adulthood. In adult females, we found no evidence of manifesting sensitisation, but a subliminal memory trace (latent sensitisation) might still facilitate responses to the subsequent injections (see Section 3.6).

### 3.4 Differences Between the Stress Paradigms

**FIGURE 5:**
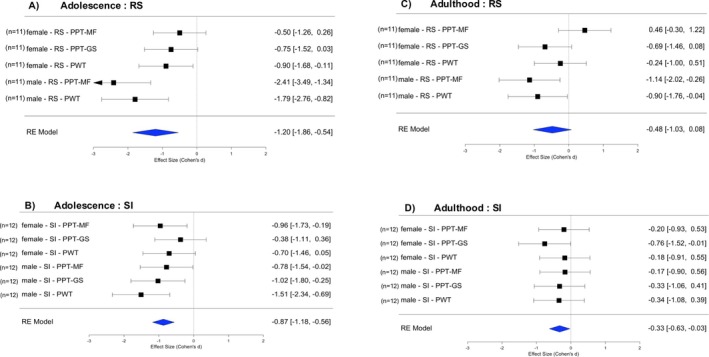
Comparison of effects of restraint stress vs. social isolation stress. Forest plots of standardised effect sizes with 95% confidence intervals across sexes and readouts (diamonds). Squares and error bars provide effect sizes calculated between stressed and respective control animal groups. Adolescence: Immediately after stress (A. RS, B. SI). Adulthood: after puberty, immediately before injections of NGF or saline (C. RS, D. SI). The male‐RS data has been obtained from Singaravelu et al. (2022). NGF, nerve growth factor (0.8 μM, 50 μL); PPT‐GS, pressure pain threshold of the lower limb gastrocnemius muscle; PPT‐MF, pressure pain threshold of the low back multifidus muscle; PWT, paw withdrawal threshold to cutaneous pin prick; RS, restraint stress; SI, social isolation stress.

Differences between the two stressors (repeated restraint RS, social isolation SI) were also evaluated using within‐study meta‐analyses of standardised effect sizes vs. the respective control group (see Forrest plots, Figure 5). Across both sexes and all three readouts, in adolescence, the effect size induced by RS was large (mean *d* = −1.20, Figure 5A) and that induced by SI was smaller but still large (mean *d* = −0.87, Figure 5B). In adulthood, the effect size was medium for RS (mean *d* = −0.48, Figure 5C) but small for SI (mean *d* = −0.331, Figure 5D). Effect sizes but also 95% CI of SI were smaller than those of RS; hence, the pooled data were significant for both stressors in adolescence but borderline not significant in adulthood.

### 3.5 Differences Between Readouts

**FIGURE 6:**
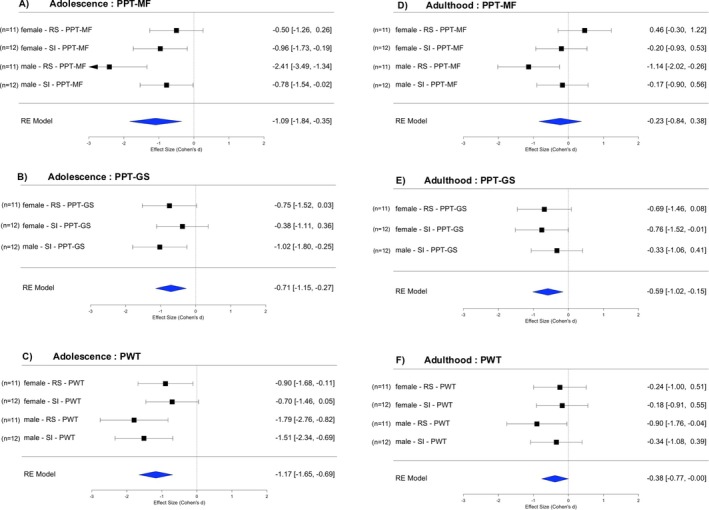
Comparison of effects of early life stress on behavioural readouts. Forrest plots of standardised effect sizes with 95% confidence intervals across stressors and sexes (diamonds). Squares and error bars provide effect sizes calculated between stressed and respective control animal groups. Adolescence: Immediately after stress (A. PPT‐MF, B. PPT‐GS, C: PWT). Adulthood: After puberty, immediately before injections of NGF or saline (D: PPT‐MF, E: PPT‐GS, F: PWT). The male‐RS data has been obtained from Singaravelu et al. (2022). NGF, Nerve growth factor (0.8 μM, 50 μL); PPT‐GS, pressure pain threshold of the lower limb gastrocnemius muscle; PPT‐MF, pressure pain threshold of the low back multifidus muscle; PWT, paw withdrawal threshold to cutaneous pin prick; RS, restraint stress; SI, social isolation stress.

Again, we subjected standardised effect sizes vs. the respective control group to a meta‐analysis (see Forrest plots in Figure 6). In adolescence, across both sexes and both stressors, the effect sizes for the PPT of the low back multifidus muscle (PPT‐MF) were large (mean *d* = −1.09, Figure 6A), for the thigh GS muscle were medium (mean *d* = −0.71, Figure 6B) and for distal cutaneous pain (PWT) were large (mean *d* = −1.17, Figure 6C). In adulthood, across the sexes and stressors, the effect sizes for PPT‐MF were small in adulthood (mean *d* = −0.23, Figure 6D), for PPT‐GS were medium (mean *d* = −0.59, Figure 6E) and for PWT were small (mean *d* = −0.38, Figure 6F). Confidence intervals of the pooled data were significant for all readouts in adolescence, right after the stress, but mostly non‐significant in adulthood. In summary, the effects of adolescent stress on the sensitivity to mechanical stimuli that evoke pain of axial and limb muscles, as well as distal hind paw skin, were pronounced immediately after stress, with incomplete recovery in adulthood.

### 3.6 Priming by Stress for Enhanced Responses to Subsequent Injections

**FIGURE 7:**
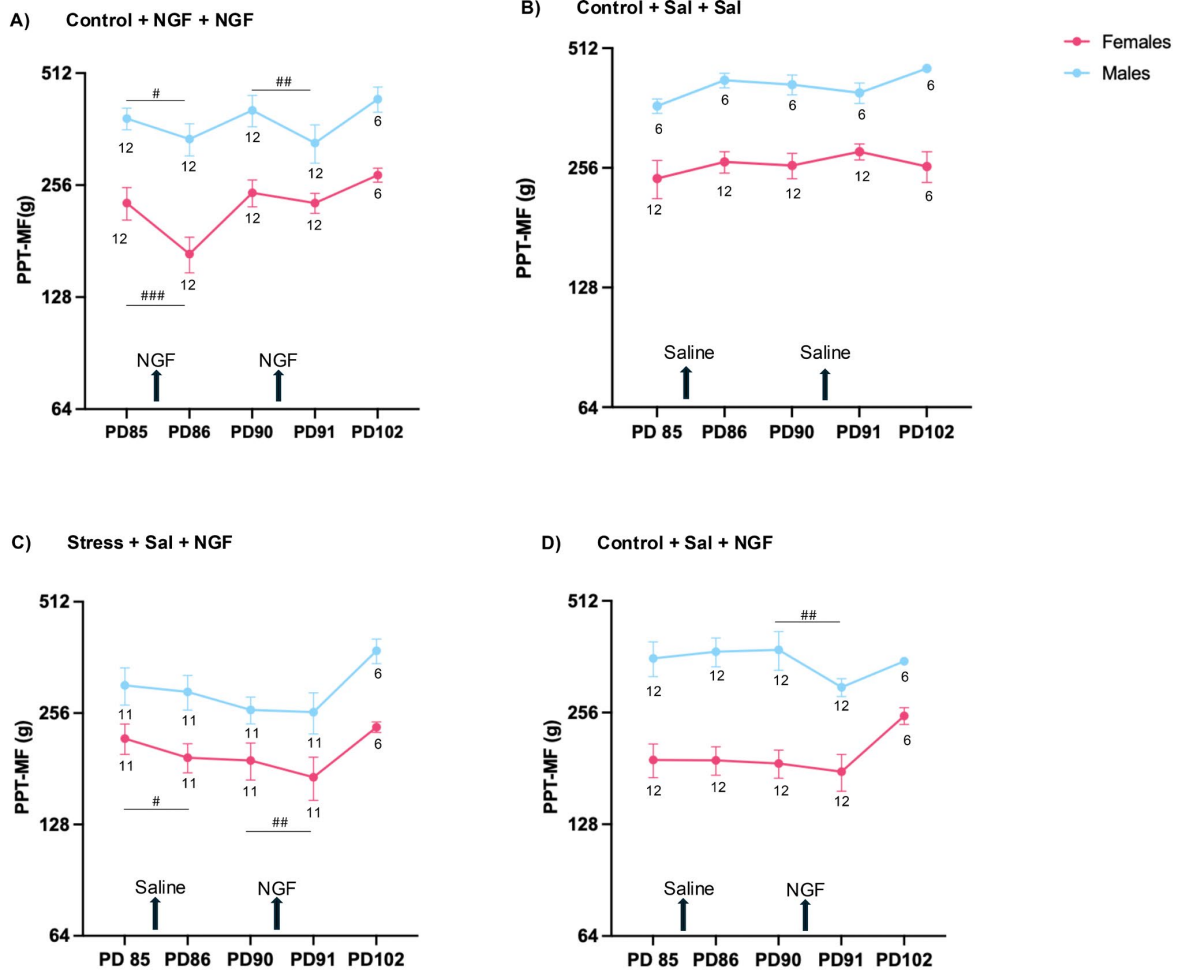
Effects of injections on low back multifidus muscle sensitivity. (A) Two NGF injections were administered consecutively on PD85 and PD90 in one group of non‐stressed animals as a positive control. Induction of a state of latent sensitisation by the first injection would have led to stronger or longer‐lasting hyperalgesia after the second injection. This was not the case in female or male Wistar Han rats. (B) Two saline injections were administered consecutively on PD85 and PD90 in another group of non‐stressed animals as a negative control for the positive control. (C) Effects of saline injection (PD85) and NGF injection (PD90) in stressed female or male Wistar Han rats (pooled across repeated restraint stress and social isolation) (D) Effects of saline injection (PD85) and NGF injection (PD90) in non‐stressed female or male Wistar Han rats (pooled controls that were only handled in the repeated restraint stress and social isolation experiments). The upward arrows represent the time point of injection administered in the ipsilateral low back multifidus muscle. Half of the male data is obtained from Singaravelu et al. (2022). The numbers mentioned in the figure around the data points represent the number of animals used for obtaining the mean value at the specific time point. Effect size: ###: *d* > 0.8 refers to a large effect, ##: 0.8 > *d* > 0.5 refers to a medium effect, #: 0.5 > *d* > 0.2 refers to a small effect size. PD, Postnatal days; PPT‐MF, Pressure Pain Threshold at the low back multifidus muscle.

According to the hypotheses derived in the introduction, we tested, using injections, whether adolescent stress had left memory traces (latent sensitisation of dorsal horn neurons) such that the effects of injections on mechanical pain sensitivities would be enhanced and longer lasting. Figure 7 illustrates mean multifidus muscle PPTs (i.e., at the injection sites) for the most salient comparisons. Effect sizes for post‐pre injection differences are listed in Table 3 (i.e., sensitisation has a negative sign).

**TABLE 3:** Effect sizes of changes in low back multifidus muscle sensitivity due to injections in conditioned and non‐conditioned groups.

Sex	Days	Group	*p*	Cohen's *d*	Group	*p*	Cohen's *d*
Female	85–86	CNN	0.051	−0.875###	CSS	0.248	0.498
Female	90–91	CNN	0.712	−0.166	CSS	0.671	0.189
Male	85–86	CNN	0.477	−0.310#	CSS	0.026	1.698
Male	90–91	CNN	0.241	−0.511##	CSS	0.699	0.280
Pooled	85–86	CNN	0.135	−0.445#	CSS	0.268	0.381
Pooled	90–91	CNN	0.272	−0.326#	CSS	0.987	−0.011
Female	85–86	SSN	0.331	−0.447#	CSN	0.932	0.047
Female	90–91	SSN	0.078	−0.568##	CSN	0.712	−0.166
Male	85–86	SSN	0.699	−0.183	CSN	0.977	0.024
Male	90–91	SSN	0.948	−0.042	CSN	0.078	−0.785##
Pooled	85–86	SSN	0.553	−0.185	CSN	0.926	−0.030
Pooled	90–91	SSN	0.584	−0.170	CSN	0.471	−0.212#

*Note:* Changes in pressure pain thresholds of the multifidus muscle before (Day 85, 90) vs. after injections (Day 86, 91) into this muscle. Group: specific injection group from both the restraint stress and social isolation, grouped together: CNN: Control + NGF + NGF; CSS: Control + Saline + Saline; SSN: Stress + Saline + NGF; CSN: Control + Saline + NGF. Left side: Conditioned groups (by preceding stress or NGF injection). Right side: non‐conditioned control groups. Pooled: data pooled across sexes. The *p*‐value is calculated using the Mann–Whitney *U*‐test: *: *p* < 0.05, **: *p* < 0.01. Cohen's *d* has been calculated using the Mann–Whitney *U*‐test statistics (Lenhard and Lenhard 2022). The effect sizes according to Cohen's d in cases where there is a threshold decrease: ###: *d* > 0.8 refers to a large effect, ##: 0.8 > *d* > 0.5 refers to a medium effect, #: 0.5 > *d* > 0.2 refers to a small effect.

As a positive control, two NGF injections separated by 5 days were administered in groups of non‐stressed animals. In previous studies on male Sprague–Dawley rats, a first NGF injection induced a transient drop in PPT and also left a memory trace of latent sensitisation that was uncovered by a second NGF injection that induced more pronounced and longer‐lasting behavioural signs of hyperalgesia (Hoheisel et al. 2013). NGF injections in male or female Wistar Han rats induced drops in PPT with small to large effect sizes (Figure 7A, Table 3), whereas control saline injections did not (Figure 7B, Table 3). When pooling across both sexes, there were no differences between NGF and saline before injection (PD85 and 90: *D* = 0.197 and 0.047), but lower PPT at the injection site 1 day after NGF (PD86, PD91) with medium to large effect sizes (*D* = 0.616, 0.634) that just missed significance vs. the saline controls (*p* = 0.057 and 0.051) (Table 4, top). While these data confirm mild acute sensitisation by low‐dose NGF in both sexes, we found no evidence for latent sensitisation by the first NGF injection in female or male Wistar Han rats.

**TABLE 4:** Effect sizes of conditioning by preceding NGF injection or stress on low back multifidus muscle sensitivity before and after injections.

Groups	Postnatal day	*p* (pooled)	*d* (pooled)	*p* (Females)	*d* (Females)	*p* (Males)	*d* (Males)
CNN vs. CSS	PD85	0.537	0.197	0.932	−0.047	0.493	0.359
	PD86	0.057	−0.616##	0.000***	−1.757###	0.179	−0.702##
	PD90	0.891	−0.047	0.513	−0.286#	0.750	−0.177
	PD91	0.051	−0.634##	0.014*	−1.139###	0.179	−0.702##
SSN vs. CSN	PD85	0.939	−0.026	0.413	0.365	0.525	−0.285#
	PD86	0.353	−0.282#	0.881	0.077	0.117	−0.708##
	PD90	0.353	−0.282#	0.739	0.154	0.026*	−1.042###
	PD91	0.299	−0.315#	0.881	−0.077	0.379	−0.392#

*Note:* Differences in pressure pain thresholds of the multifidus muscles between conditioned (by preceding NGF injection or stress) vs. non‐conditioned groups: CNN: Control + NGF + NGF; CSS: Control + Saline + Saline; SSN: Stress + Saline + NGF; CSN: Control + Saline + NGF. PD85 and PD90 were before injection, PD86 and PD91 after injection into this muscle. Pooled: data pooled across sexes. Cohen's *d* has been calculated using the Mann–Whitney *U*‐test statistics (Lenhard and Lenhard 2022). The *p*‐value is calculated using the Mann–Whitney *U*‐test: *: *p* < 0.05, **: *p* < 0.01, ***: *p* < 0.001. The effect sizes according to Cohen's *d* in cases where there is a threshold decrease: ###: *d* > 0.8 refers to a large effect, ##: 0.8 > *d* > 0.5 refers to a medium effect, #: 0.5 > *d* > 0.2 refers to a small effect.

In stressed female Wistar Han rats (pooled across RS and SI, Figure 7C), a saline injection on PD85 led to a small drop in PPT (Cohen's *d* = −0.447, *p* > 0.05) that did not recover within 5 days, while no such effect was seen in males (Cohen's *d* = −0.183). Subsequent NGF injection in females on PD90 led to a medium‐sized drop in PPT (Cohen's *d* = −0.568), while in males, again, there was no effect (Cohen's *d* = −0.042). In two other groups of stressed females that received two saline injections, a saline injection on PD85 likewise led to a drop in PPT that was medium‐sized (Cohen's *d* = −0.617), while again no such effect was seen in males (Cohen's *d* = −0.118; data not shown). We conclude that adolescent stress had left a mild latent sensitisation in females, making them susceptible to sensitisation by the mechanical stimulation of an intramuscular saline injection in adulthood. The lack of evidence for latent sensitisation in males may be a floor effect due to the persistence of the manifest sensitisation by stress into adulthood (Figure 2B).

In non‐stressed female Wistar Han rats, there were no changes in pain sensitivity after the first injection (saline) or second injection (NGF, Figure 7D). However, males showed a medium‐sized effect post‐NGF (Cohen's *d* = −0.785, *p* > 0.05), reflecting the typical acute effects of this dose of intramuscular NGF.

When pooling across both sexes (Table 4, bottom), there were no differences between stress and control before the first injection (PD85: *D* = 0.026), but lower PPT at the injection site with small to medium effect sizes in stressed animals 1 day after the first injection (saline, PD86: *D* = −0.282), 5 days after this injection (PD90: *D* = −0.282) and 1 day after the second injection (NGF, PD91: *D* = −0.315). On PD102, there was again no difference between stress and control (*D* = 0.071; data available for the SI only). As illustrated by comparing Figure 7C,D, these differences appear to reflect enhanced responsiveness in stressed females to the mechanical effects of injections (to both saline and NGF). In stressed males, these differences appear to reflect a persistent manifest sensitisation by adolescent stress.

## 4 Discussion

We found that early‐life physical trauma (restraint stress) led to behavioural signs of sensitisation of dorsal horn neurons, more pronounced and longer‐lasting in male than female Wistar rats. Emotional neglect (social isolation) also induced more pronounced signs of dorsal horn sensitisation in males than in females. Meta‐analysis indicated that smaller effect sizes after social isolation are partly compensated by smaller confidence intervals and better homogeneity across sexes and readouts. Sensitivity to change was lower for the PPT of the gastrocnemius than for the multifidus muscles and for paw withdrawal to pinprick.

These data suggest that adolescent stress can induce widespread manifest spinal sensitisation to both deep tissue and cutaneous inputs, persisting into adulthood in males but not females. Mildly enhanced responses to subsequent saline injections (mechanical challenge) suggest a subliminal memory trace (latent sensitisation) in adult females but not males. The myofascial low back pain model of two NGF injections 5 days apart that had previously been established in Sprague–Dawley rats did not produce latent sensitisation in Wistar‐Han rats.

### 4.1 Mechanical Hypersensitivity in Stress‐Induced Myofascial Low Back Pain

In male Sprague–Dawley rats stressed in adulthood, nociceptive dorsal horn neurons increased their resting activity and developed new receptive fields in deep tissues of the hindlimb with unchanged input from the skin (Hoheisel and Mense 2015). These findings predicted that stress‐induced ongoing low back pain and deep tissue hypersensitivity may radiate pseudoradicularly into the lower limb. Human studies with cpLBP have demonstrated that patients with childhood adversities exhibited increased deep tissue pressure pain sensitivity (Tesarz et al. 2016). In our animal model, stress during adolescence induced deep tissue hypersensitivity, while in our previous study with adulthood stress, the PPT of the low back remained unchanged (Hoheisel et al. 2015). These findings confirm the relevance of vulnerable periods during childhood and puberty for the development of cpLBP (Bussières et al. 2023; Dalechek et al. 2024; Thomas and Goodin 2022). Contrary to the prediction, mechanical hypersensitivity was not restricted to deep tissues; mechanical hypersensitivity for hindpaw skin resembles characteristics of chronic widespread pain and fibromyalgia in humans (Blumenstiel et al. 2011).

### 4.2 Differences Between Emotional Neglect and Physical Trauma

Restraint stress paradigm induces long‐lasting hyperalgesia (Gameiro et al. 2005; Li et al. 2016; Scheich et al. 2017) and may be considered a rodent surrogate model for childhood physical trauma in humans (abuse). The social isolation paradigm enhances somatic pain sensitivity that can be prevented by environmental enrichment (Filaretova et al. 2024). It may be considered a rodent surrogate model for childhood emotional stress (Bungert et al. 2015). In humans, physical violence predisposes aggression‐related conditions (Bounoua et al. 2020), while emotional neglect predisposes depression or metabolic syndromes (Lee et al. 2014). We found that repeated restraint stress exerted a larger physiological effect than social isolation, although social isolation was applied for longer (29 days) than restraint stress (12 days). (Zhou et al. 2022) found hypersensitivity after 28 days and analgesia after 56 days of social isolation, indicating habituation limits its effects.

### 4.3 Sex Differences in Susceptibility to Stress‐Induced Myofascial Low Back Pain

Females are usually more sensitive to painful stimuli (“evoked pain”) than males (Francis‐Malavé et al. 2023; Mogil 2012; Wei et al. 2022), potentially due to hormonal differences (Smith 2019). This is true for evoked pain in humans, as demonstrated by Quantitative Sensory Testing (Rolke et al. 2006). Musculoskeletal pain conditions (“ongoing pain”) tend to be more frequent in females than males, as reported in cpLBP (Overstreet et al. 2023), fibromyalgia (Jurado‐Priego et al. 2024) and temporomandibular disorders (Khan et al. 2024). Therefore, the current findings that males exhibited longer‐lasting manifest sensitisation after adolescent stress than females were unexpected.

Some previous studies have found greater pain sensitivity after stressors in males than females (Mogil 2012). Neonatal exposure to painful stimuli (hind‐paw incision injury in rats, heel lancing in humans) leads to stronger hypersensitivity in males later in life (Beggs et al. 2012). These findings have been interpreted to reflect the generation of persistent memory traces. In the NGF model of cpLBP, subliminal memory traces (latent sensitisation) are established by the first NGF injection that primes dorsal horn neurons to be more responsive to the second NGF injection (Hoheisel et al. 2013). We predicted that adolescent stress would leave such a subliminal memory trace into adulthood. In females, we found some evidence for latent sensitisation, as a mild mechanical stimulus (intramuscular saline injection) induced a mild but long‐lasting muscle hypersensitivity. In males, long‐lasting manifest sensitisation for mechanical inputs may have occluded any signs of latent sensitisation. In aggregate, stressed males exhibited pronounced manifest sensitisation into adulthood, while adult females seemed to have recovered but remained susceptible to sensitisation by mild muscle insults (Shaw et al. 2020). Such differential stress sensitivity may contribute to the higher prevalence of cpLBP in females.

### 4.4 Strain Differences in Myofascial Low Back Pain Models

Wistar rats were used in this study because they are more prone to anxiety‐like behaviour in stress studies (McAuley et al. 2009). Wistar rats tend to be more sensitive to pain than Sprague Dawley (SD) rats (Hestehave et al. 2019; Taylor et al. 2001). Our data support the usage of Wistar rats to study stress as a risk factor for myofascial pain, as we saw manifest sensitisation into adulthood, while a previous study in SD rats did not report changes in behaviour (Hoheisel et al. 2015).

In contrast, the latent sensitisation model (two NGF injections, 5 days apart) established in SD rats (Hoheisel et al. 2013) did not apply to Wistar rats as the second injection caused no greater or prolonged hypersensitivity than the first. This may be explained by stronger habituation to test–retest settings in Wistar rats that has also been documented in auditory startle reflexes (Hestehave et al. 2019; Pilz et al. 1999).

### 4.5 Priming by Stress for Dorsal Horn Neuron Sensitisation

The ability of saline injections to induce local mechanical hypersensitivity in stressed female animals suggests that stressful events in adolescence left some subliminal memory trace in the dorsal horn (Smith et al. 2021). Musculoskeletal pain was weaker than previously reported in female SD rats (Syrett et al. 2022). Wistar rats may be less prone to sensitisation by nociceptive stimuli as they also exhibit weaker responses to injection of complete Freund's adjuvant than other strains (Hestehave et al. 2019).

Latent sensitisation by nerve growth factor (NGF) is attributed to enhanced postsynaptic dorsal horn neuron sensitivity, potentially through activation of fractalkine signalling pathways involving microglia (Sessler et al. 2021). However, here, NGF failed to induce long‐lasting sensitisation in control and stressed animals. This may be due to bidirectional effects of NGF injections. In addition to direct spinal sensitising effects (Curatolo 2024; Hoheisel et al. 2007), noxious stimuli such as NGF can activate indirect top‐down modulation that is mediated via the brainstem and may be inhibitory or excitatory (Le Bars et al. 1979; Patel et al. 2024). The brainstem is critical in setting long‐term balance between pain‐enhancing and pain‐reducing systems. Descending inhibitory controls are insufficient to suppress central sensitisation induced by noxious stimuli (Patel et al. 2024). However, metaplasticity of brainstem controls might shift the balance of excitatory and inhibitory processes in the dorsal horn region.

Metaplasticity is the activity‐dependent modulation of synaptic plasticity (Abraham 2008). Stress can reduce long‐term potentiation (LTP) and enhance long‐term depression (LTD) in the hippocampus (Artola et al. 2006) and in spinal nociceptive circuits (Li and Baccei 2016). Thus, stressed females may exhibit enhanced LTD in response to NGF injections, which could suppress NGF‐induced spinal sensitisation. These findings align with previous reports that adolescent stress can cause long‐lasting, sex‐specific alterations in pain modulation pathways, favouring inhibition in females and resilience in males (Li and Baccei 2016).

### 4.6 Strengths and Limitations

A strength of our study is the use of multiple behaviour readouts (axial/distal, muscle/skin), demonstrating widespread pain in animals. The three readouts also increased statistical power in a within‐study meta‐analysis. Limitations include no assessment of the emotional aspects of pain, potential social transfer of learned effects of stress to control animals and interactions with stress due to transportation after weaning. Some data were reused from a previous study (Singaravelu et al. 2022), and the study design lacked three groups needed for a full factorial model. These limitations were intentional to align with the 3R principles, but we cannot exclude that subliminal latent sensitisation by both stress and first NGF would only show as manifest prolonged sensitisation when combined in the same animals.

### 4.7 Summary and Conclusion

Both early life stress models induced immediate widespread muscular and cutaneous hypersensitivity that tended to recover towards adulthood. Compared with previous studies in Sprague–Dawley rats, Wistar Han rats were more susceptible to spinal sensitisation by stress but less so by nociceptive input. Males were more susceptible to long‐lasting manifest sensitisation by stress than females, whereas females exhibited some evidence for latent sensitisation, causing hypersensitivity upon a second hit in adulthood (injections into multifidus muscle). The differential stress sensitivity may contribute to the higher prevalence of cpLBP in females.

## Author Contributions

This study was designed by D.S., W.G. and R.‐D.T. The experiments were performed by D.S. with support by L.L. The blinding was done by L.L. The data were analysed by D.S. and R.‐D.T., and the results were critically examined by all authors. D.S. had the primary role in preparing the manuscript, which was edited by W.G. and R.‐D.T. All authors have approved the final version of the manuscript, ensuring its accuracy and completeness in all aspects of the work.

## Conflicts of Interest

The authors declare no conflicts of interest.
